# A multimodal endoscopic approach for esophageal fistula closure

**DOI:** 10.1055/a-2436-1041

**Published:** 2024-11-13

**Authors:** Andrea Telese, Benjamin Norton, Apostolis Papaefthymiou, Alberto Murino, Charles Murray, Rehan Haidry

**Affiliations:** 1 591481 Digestive Disease and Surgery Institute, Cleveland Clinic London, London, United Kingdom of Great Britain and Northern Ireland; 2 Division of Surgery and Interventional Science, University College London, London, United Kingdom of Great Britain and Northern Ireland; 3 Department of Medicine, University College London Centre for Obesity Research, London, United Kingdom of Great Britain and Northern Ireland

Several endoscopic options are available for the treatment of post-surgical fistulae including clipping, stenting, vacuum therapy, and endoscopic suturing. Choosing between these depends on multiple factors including defect size and site, etiology, and presence of associated cavity 1 2 3 . We present the case of a 59-year-old man with recurrent chest infections secondary to an esophago-jejunal anastomotic fistula. He had previously undergone a total gastrectomy and chemotherapy for gastric adenocarcinoma (T4aN1M1), followed by distal pancreatectomy, hepatic resection, and jejunal repair for primary pancreatic adenocarcinoma (T4aNxMx; R1) that left him with an esophago-jejunal anastomosis. He continued adjuvant chemo-immunotherapy; two years later he had evidence of progressive disease and developed respiratory symptoms.

Our endoscopic assessment demonstrated a 5–10 mm transmural anastomotic defect. The fistula was initially treated with argon plasma coagulation (FiAPC probe; Erbe Elektromedizin, Tübingen, Germany) and endoscopic suturing (OverStitch Sx; Apollo Endosurgery, Austin, Texas, USA) and an over-the-scope clip (OTS clip; Ovesco Endoscopy, Tübingen, Germany) to ensure closure. As respiratory symptoms persisted with evidence of recurrent fistula on barium imaging, a decision was made to place a vacuum-stent (VACStent GI; Möller Medical, Fulda, Germany) over a guidewire (Jagwire; Boston Scientific, Marlborough, Massachusetts, USA) under direct vision to cover the defect ( Video 1 ). Following this, oral intake was gradually restored and the vacuum-stent was removed on the seventh day uneventfully using a distal attachment cap (DH-28GR Hood; Fujifilm, Tokyo, Japan) and a grasping device (Raptor; Steris, Mentor, Ohio, USA). Abundant granular tissue was noted and dynamic on-table esophagogram showed no leakage. Fistula closure allowed continuation of adjuvant therapy. After this treatment, respiratory symptoms instigated a barium swallow that showed fistula recurrence, and another vacuum-stent was placed under fluoroscopy ( Fig. 1 ). The following day, a decision was made with the patient to continue vacuum-stent therapy at home for seven days. At removal, granulation tissue was present, and despite a suspicion of a small defect, dynamic on-table esophagogram showed no contrast leak. After vacuum-stent removal, the patient remained asymptomatic for fistula-related symptoms and a barium swallow showed no fistula recurrence. The patient continued his chemotherapy and a subsequent CT scan while asymptomatic for fistula-related symptoms demonstrated a possible fistulous tract ending in an atelectatic cavity. A later endoscopy with on-table esophagogram could not identify any fistula and no other fistula-related treatment was pursued. In the following follow-up the general status of the patient deteriorated but no other fistula-related intervention was required.

**Fig. 1:**
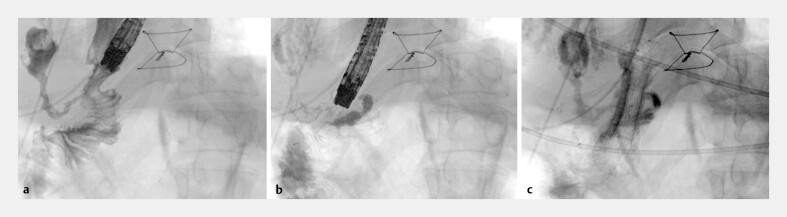
Fluoroscopic images. **a** Recurrence of anastomotic fistula following first vacuum-stent removal. **b** Submucosal radiopaque marking for second vacuum-stent placement. **c** Second vacuum-stent after deployment.

Multimodal endoscopic approach for esophageal fistula closure.Video 1

A vacuum-stent can be an effective treatment of post-surgical fistula, provided the right support and infrastructure, and the vacuum-stent can be used as a community-based therapy to reduce the length of admission.

Endoscopy_UCTN_Code_TTT_1AO_2AI

## Notes

Endoscopy E-Videos https://eref.thieme.de/e-videos
*E-Videos* is an open access online section of the journal *Endoscopy* , reporting on interesting cases and new techniques in gastroenterological endoscopy. All papers include a high-quality video and are published with a Creative Commons CC-BY license. Endoscopy E-Videos qualify for HINARI discounts and waivers and eligibility is automatically checked during the submission process. We grant 100% waivers to articles whose corresponding authors are based in Group A countries and 50% waivers to those who are based in Group B countries as classified by Research4Life (see: https://www.research4life.org/access/eligibility/ ). This section has its own submission website at https://mc.manuscriptcentral.com/e-videos .
